# The Social Triad Model: Considering the Deployer in a Novel Approach to Trust in Human–Robot Interaction

**DOI:** 10.1007/s12369-023-01048-3

**Published:** 2023-09-13

**Authors:** David Cameron, Emily C. Collins, Stevienna de Saille, Iveta Eimontaite, Alice Greenwood, James Law

**Affiliations:** 1 Information School, University of Sheffield, Sheffield, S10 2TN UK; 2 Institute of Experiential Robotics, Northeastern University, Boston, MA 02115 USA; 3 Department of Sociological Studies, University of Sheffield, Sheffield, S10 2TN UK; 4 School of Aerospace, Transport and Manufacturing, Cranfield University, Bedford, MK43 0AL UK; 5 Department of Computer Science, University of Sheffield, Sheffield, S10 2TN UK

**Keywords:** Trust, Human–human interaction, Transparency, Deployer

## Abstract

There is an increasing interest in considering, measuring, and implementing trust in human-robot interaction (HRI). New avenues in this field include identifying social means for robots to influence trust, and identifying social aspects of trust such as a perceptions of robots’ integrity, sincerity or even benevolence. However, questions remain regarding robots’ authenticity in obtaining trust through social means and their capacity to increase such experiences through social interaction with users. We propose that the dyadic model of HRI misses a key complexity: a robot’s trustworthiness may be contingent on the user’s relationship with, and opinion of, the individual or organisation deploying the robot (termed here, Deployer). We present a case study in three parts on researching HRI and a LEGO^®^ Serious^®^ Play focus group on care robotics to indicate how Users’ trust towards the Deployer can affect trust towards robots and robotic research. Our Social Triad model (User, Robot, Deployer) offers novel avenues for exploring trust in a social context.

**Supplementary Information:**

The online version contains supplementary material available at 10.1007/s12369-023-01048-3.

## Introduction

The increasing ubiquity of autonomous robotics and AI in daily life can be seen across the globe. National policies, such as the UK’s Industrial Strategy and RAS 2020 highlight the impact robotics and autonomous systems (RAS) are expected to have on socio-economic structures across many settings. Moreover, substantial strategic funding has been invested to form research networks such as Trustworthy Autonomous Systems,^1^ to address questions of user-interaction within wider socio-technical contexts of how to build and deploy systems that people can trust. In this paper we explore how the social contexts in researching even seemingly straightforward human-robot interaction (HRI) scenarios may shape the course, and potentially outcomes, of HRI studies. We consider, how do the wider relationships in an HRI study influence the measurements of trust around which trustworthy HRI studies are formed, and analysed?

Two cornerstones of social robotics research are the simulation of social processes in robotic agents and the study of people’s social processing and experiences regarding HRI [1]; collectively, the field highlights where psychological phenomena may have relevance in developing robots for HRI. Examples may include robots using various social-like behaviours to influence user trust [2], and the applicability of socio-cognitive models [3–5] to measure and explain trust in HRI in terms of cognitive and social components.

The exploration of trust as having a social aspect is still a relatively new area of study for HRI. While established models of trust such as the Human, Robot, and Environment factors [6–8] cover a lot of ground, trust towards robots has been largely explored in terms of cognitive factors such as beliefs of reliability or capability [9–13]. Emerging social models draw from the burgeoning literature on various simulated social strategies used in HRI to influence users’ trust, (e.g., persuasion [14], expression [15], apologies [16, 17], and promises [18]) to argue that the social aspect of trust seen in human-human interaction [3, 5] has relevance in HRI [16, 19]. However, sizeable variation in measurement and definition of what constitutes social trust affects the emerging field [20], and clarity in these would benefit further predictive models for trust [21, 22].

While we do not aim to resolve this here, collecting measures of social trust, and research using these, brings about an apparent contradiction: though trust may be fruitfully explored in social terms in HRI, it is not always apparent towards whom (or what) the trust is directed. A robotic agent involved in an interaction, whether explicitly designated a ‘social robot’ or not, remains part of the mediated communication between it and the humans around it. In having a presence that influences a humans’ social interaction, any robot could be considered within socio-emotional trust frameworks.

In this paper, we first highlight this contradiction in the literature; second, we propose a model in attempt to resolve this; third, we provide examples in the form of a case study on conducting HRI research and a focus group on care robotics; and lastly, we offer avenues for exploring trust’s social aspect in HRI.

### Measuring Trust’s Social Context in HRI

Measuring trust in HRI has historically been achieved through importing or adapting early scales that measure trust in automation [23, 24]; more recent work builds on these to target HRI specifically [25, 26]. Although these scales consider trust in terms of the physicality of the systems and their reliability or predictability, there are glimmers of trust’s social aspect. These include: identifying potential loss of trust after being ‘betrayed’ [23, 25] [p. 236], recognition that ‘people do not perceive concepts of trust differently across different types [i.e., human or machine] of relationships’ [24] [p. 31], use of socially relevant items such as ‘the system is deceptive’ and ‘the system behaves in an underhand manner’ [27], and consideration of components that make reference to social aspects such as ‘Most robots are [caring/friendly/kind] towards people’ [26] [pp. 88–91].

Each of the above resemble ways in which trust has been conceptualised in human-human interaction, (e.g., relating to morality, benevolence, warmth [3, 5, 28]). Emerging work, such as the Multi-Dimensional Measure of Trust (MDMT) [29], has embraced the idea that conceptions of social trust in human-human interaction may benefit trust research in HRI and put forward that users evaluate a robot’s trustworthiness along multiple aspects. The precise nature of these are still very much up for debate (e.g., capability, integrity and deceit [16, 30] or being reliable, capable, sincere and ethical [19, 29]). However, as with psychological models of trust, the broad strokes of a cognitive (e.g., reliability) and affective (e.g., benevolence) dimensions are considered [4].

A hurdle to address in the creation and use of social trust measures for HRI is establishing whether social trust is particular to social robotics (where one might imagine robot carers, teachers, service workers etc...) or further applies to interactions with robots that are not explicitly social (e.g., co-botics in manufacturing). Moreover, the widespread successful use of scales examining trust as reliability suggest common understanding of trust towards robots in this capacity, it still remains to be seen if this occurs for social trust.

### Challenges in Measuring Trust’s Social Context

Recent work using the MDMT [19] highlights challenges in exploring social trust in HRI, arising from people’s beliefs on robots’ capacities to be social [31]. Specifically, participants at times select ‘does not apply’ for the more social measures of trust, especially if the hypothetical robot did not appear to be overtly social (i.e., not humanoid and/or not using synthetic speech).^2^ Of particular importance to the current work, participants justify this decision by framing the robot as being without agency and/or serving another agent *external to the interaction*.

Similarly, individuals’ assignment of responsibility highlights views of a robot’s dependence on external agents. Individuals evaluating moral decision scenarios assign more blame towards a robot for its error than a human making the same error [33], but if blame can also be assigned at a broader level, it is shifted externally for the robot only (i.e. towards a robot’s owner but not towards a human’s manager) [34]. This suggests that the external agents deploying the robot are not seen as wholly separated from the robot.

Clark and Fischer argue that the external human agent is less external than ordinarily characterised in robotics research [35]: social robots still exist in viewers’ imagination alongside ventriloquist dummies or puppets. In their words, ‘[p]eople conceive of social robots... not as social agents per se, but as depictions of social agents’ [p. 26]. They put forward that people recognise three classes of agents in HRI: the character (depicted in the robot), the audience (the users/themselves) and the authority (the often unseen person/s deploying the robot) [35]. We argue that it is within this context of three agents, rather than two, that social trust may be best understood.

While trust towards the agents deploying the robot has been mentioned before [36], this role is still not well explored. This may be due to presumptions about the deploying agents’ intent: ‘it is hard to imagine... developers did not act in a benevolent manner’ [p. 7]. Rather than discarding the deploying agents’ influence entirely [36], users’ beliefs of the deploying agents’ trustworthiness may affect trust towards their robot, and have relevance for HRI research on trust. For example, a user may distrust the motives of their employer for deploying a robot into the workplace, manifesting in HRI as distrust of the robot itself.

In sum, we propose that individuals assess trust towards a robot *within* a human-human interaction social context (i.e., trust towards the deploying agent), seemingly peripheral to the HRI scenario underway. Exploring trust in HRI from a social aspect might then not be confined to research on social robotics; indeed, additional simulation of social interaction from such robots may serve to muddy this wider context. To develop a model of how trust within the social triad of *The User* (audience), *The Robot* (character), and *The Deployer* (authority) (cf. [35]) may operate, we draw from Vicente’s model of the Tech Ladder [37] to draw out the Deployer’s role in HRI.

### HRI Across Multiple Levels

As previously argued, documentation of factors affecting trust in HRI is not considered exhaustive [8]. The Tech Ladder presents five levels, progressively broadening from the physical object (here, a robot) to the social contexts of deployment, such as regulation [37]. Table 1 presents the five levels of the Tech Ladder in relation to robots for HRI.

**Table 1:** Levels of HRI within the Tech Ladder

Tech Ladder level	Representation in HRI
5. Political (regulation and oversight)	Allocation of resources, legislation for safety and useage in proposed context
4. Organizational (motivations for deploying the robot)	Reasons for deployment. The context for, and bounds of, the specific interaction
3. Team (co-botics/cooperative interaction)	Compatibility of goals, task delegation, authority in decision making, sequences in interaction
2. Psychological (inferences of the robot’s processing and action)	The robot’s reliability, information communicated, apparent goals. User’s experience with robotics
1. Physical (the robot itself)	The robot’s morphology, modes of communication capacity to navigate or affect the environment

#### Trust at the Lower Levels

Trust towards a robot at the Physical and Psychological levels are well documented in the field and represented in reviews as various distinct factors [8], including morphology [38], proximity [39], communication modality [40], and reliability [11]. Psychological factors would also extend to users’ experience with the system [41], their identifying predictable behaviours [42], and allocation of attention towards monitoring behaviour [9].

#### Trust at the Team Level

Robots are increasingly deployed in interaction roles as teammates [17] and the capacity for robots to collaborate with humans, is anticipated to become more important for interaction [43]. At this level, a social context may be apparent where robot communication and behaviours imply agency, personality [44] and/or a social role [45].

As with human-human teams, simulated socially interactive behaviours can shape trust towards the robot [14–18]. Recent research on trust in HRI puts forward that such outcomes indicate a dimension of social trust in HRI [30, 46, 47] (though diverge in the specifics), although an opposing view argues that as robots are incapable of experiencing benevolence or integrity [43] such measures may be irrelevant or misleading. Nonetheless, HRI offers unique interaction circumstances that simultaneously present the robot as a device and as an agent; it is precisely this liminal nature that encourages people to make cognitive inferences which assign intentionality [48] or social norms [49] to the robot, and then respond *as if* it is an independent agent despite understanding its lack of independence from the Deployer [31, 35]. In other words, trust at the team level is social, but social towards whom?

#### Trust at the Higher Levels

The ambiguity of trust in HRI seen at the Team Level points towards an outside influence more clearly seen at higher levels. Where robots are said to lack agency or intentions, operating only as a product of others’ intentions [31, 43], the agent deploying the robot is generally considered as being responsible [34]. Although this agent might not directly engage in an HRI scenario, their motivations at the Organizational or Political levels for use of the robot may shape HRI.

Examples of direct influence from the Deployer to shape users’ trust towards robots are still comparatively few, and given the variety of robotic systems and areas for deployment - from robots as a research tool [50], to providers of comfort [51, 52], to replacements of the workforce [53, 54] - there will be a corresponding variety of motivations. Organizational efforts to shape trust towards robotics focus on addressing the employees’ emotional experience rather than adjusting the robot or HRI scenario itself [55], and include use of ‘internal top-down communication strategies’ [56] [p. 697] to promote affective trust in organizations intending to introduce robotics.

Additionally, at the Political level, questions of who should be held liable for autonomous agents’ actions [57–59] and what policies for responsible deployment might look like [60] are still widely debated. Such questions are far beyond the scope of this paper, but understanding where persons engaging in HRI *believe* responsibility lies could fruitfully direct research attention towards trust within the social contexts which are critical to these debates. In this sense, it is useful to consider both *how the context came to be* and *who determines the interaction context*.

## The Social Triad of HRI

As an answer to the challenges faced in researching trust in a social context, we propose that ‘HRI’ as studied - interaction between user and robot in a specified scenario - reliably explores trust in terms of the Tech-Ladder’s Lower, and potentially, Team Levels. However, solely examining that interaction marginalises the influence that higher levels have on the ‘shape’ of the scenario and the interaction experience itself. While a person deploying a robot might consider themselves to be external to the HRI scenario created, their influence, via these higher levels, could still be experienced by the user and is therefore vital to capture.

**Fig. 1:**
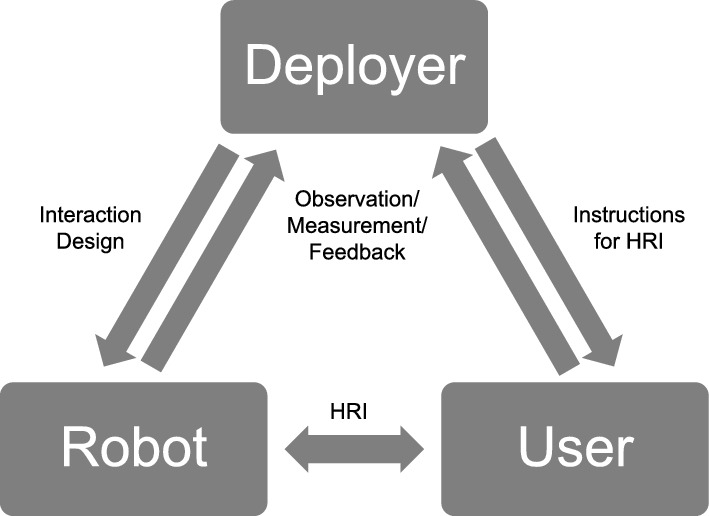
Pathways of communication for an HRI scenario

Our model (Fig. 1) seeks to explicitly include the role that these ‘external’ agents may have, and their impact on trust in a specific interaction. We draw from the apparent ambiguity of a robot as both seemingly an independent agent and connected to an authority agent [31, 35] to expand the model of HRI as a social dyad between User and Robot into a social *triad* that includes the Deployer as an interested external agent (such as a researcher, manager or corporation). It is this agent whose actions and relationship with the User shapes the HRI scenario, as they are responsible both for the Robot and for enabling the interaction scenario to occur in the form it does.

### Interactions in the Triad

HRI research thoroughly documents the (often reciprocal) interaction between User and Robot; given the high attention paid to these interaction paths, we refer to these as ‘Measured HRI’. Within an HRI scenario, users might have influence over a robot’s actions through their own behaviour, such as direct manipulation [61], remote control [62], or social/emotional expression [63]. In return, users’ experiences of the interaction may be influenced by the robot’s actions, including social-like expressions and behaviours [14–18].

The interction from Deployer to Robot is also well-considered in HRI, including: directly controlling robot behaviours [64]; specifying goals, or developing architectures to generate specific behaviours [65, 66]. In the social triad, programming the robot is not necessarily a core aspect of the role; rather, the Deployer is the agent who the User views as *being responsible for the robot* (cf. the authority [35]). Responsibility may include (but is not necessarily limited to) ownership of the robot, determining the scenario for the robot’s use, and specifying the boundaries for Measured HRI.

The interaction from Deployer to User sets out the social and applied context for the Measured HRI scenario [67]. This can include expectations and instructions for the interaction with the robot, and information on any measures used. These may be directly communicated, such as information that researchers provide participants or that a manager might provide an employee. Ahead of any Measured HRI, Users may have made evaluations of the Deployer’s trustworthiness, shaping the progression of Measured HRI. The impact of this under-explored but important aspect of the social triad is examined in Sects. 3 and 4. An absent Deployer may also create a social context to shape Measured HRI—for example, unstructured interaction environments with no obvious Deployer (i.e., authority [35]) can enable transgressive behaviour towards the robot [68].

The remaining interaction pathways refer to the Deployer receiving information from the User and Robot. Feedback on Measured HRI may be gathered passively through Deployer observation or actively through agreed feedback mechanisms (e.g., in research contexts, questionnaires or interviews; in industrial deployment, performance appraisals etc.) Simultaneously, the robot might share information on the interaction with the Deployer, again through a Deployer observing its behaviour, or through active sharing of its states or of information gathered from the User (e.g., voice recordings [69], movement [70], emotional expressions [71] etc.). User trust towards the Deployer with regard to transparency both of the data collected and its potential use may also shape trust towards the Robot as the mechanism for that data collection [72].

In sum, HRI scenarios requiring a User trust a Robot also requires trust towards the Deployer. A User may be able to evaluate the Robot itself for some aspects of trust such as reliability (e.g., [12]); however, determining social aspects of trust (such as benevolence) may further reflect User evaluations of the Deployer’s trustworthiness in this regard. Simulated benevolence from a Robot may reflect - or mask - the intentions behind its deployment and current approaches in Measured HRI have limited capacity to draw out this aspect when focusing on trust towards a robot [31]. In the following sections we present instances where trust towards a Deployer shapes the development and progression of HRI.

## Deployer Case Study

Within HRI research, the Deployer’s potential influence on studies viewed in isolation may not be obvious. However, by comparing studies we highlight the impact the Deployer may have in relation to the studies’ progression. We provide an overarching case study across three HRI experiments, where the interaction scenario and the Measured HRI element are similar enough for productive comparison to illuminate the Deployers’ influence.

All three studies used an abstracted manufacturing scenario to examine the influence of robot-supplied graphical information on the confidence and ability of users. All studies followed the same interaction procedure: participants used a KUKA iiwa collaborative robot arm to extract bolts from a set of narrow transparent tubes positioned between user and robot. The arm was pre-programmed with the tube locations but not with information about which tubes contained bolts: these could only be perceived by the operator [73]. To complete the task, participants directly manipulated the arm over each bolt to be retrieved; the arm would refine its position to the nearest tube, use a long magnetic tool to collect the bolt, and return it to the participant [74]. Timing and accuracy were monitored so that errors (e.g., moving the arm too quickly or out of range) would trigger an automatic safety stop on the robot, requiring the experimenter to reset the system. Ethical approval for each of the studies was obtained from the Department of Psychology at The University of Sheffield ahead of recruitment.

The primary interest in these studies for this paper is not the experimental outcomes (of ISO-style graphical signage on performance), which have been reported elsewhere [61, 75],^3^ but rather the differences in populations for the studies, their relations to the Deployers, and how the studies progressed. We present this as a case study in three parts where participants were: (1) university staff and students (2) non-unionised manufacturing workforce for High-Volume Low-Value components and (3) unionised manufacturing workforce for High-Value Low-Volume.

### University Research

This was conducted at the host university [61] and participants were recruited through the opt-in volunteers list; the study included the opportunity to win one of five £10 Amazon vouchers, irrespective of task performance. In general, participants were familiar with the processes of experimental research - though not necessarily the scenario or HRI - and familiar with the university’s procedures for providing informed consent.

Participants saw little risk in participation and minimal concerns were raised regarding the use of data or their anonymity. People’s spontaneous self-reported motivation for participation included: interest in research, interest in robotics, and enjoyment in helping the university community. Given the task differed substantially from participants’ current studies or (largely clerical) occupations, there was little potential for performance in the task to meaningfully reflect on anything outside of the study itself. In other words, there was no obvious route by which a negative outcome could occur from participation and thus little reason to distrust the researchers as the Deployers for the interaction. Moreover, as participants and research team were from the same institution, this commonality (i.e., ingroups) may have further prompted trust in the Deployers.

In this instance, the study situated the researchers as dispassionate and ultimately benign in their influence on the study’s progression or outcomes. One of the main drawbacks, however, is the somewhat artificial environment created; people with no meaningful reason to be using a KUKA iiwa in their daily lives were invited to participate in a robotic manufacturing scenario, staged in a non-manufacturing/laboratory environment. Though these may be elementary points, they nonetheless could meaningfully shape how people approached the HRI scenario; outcomes from the work might not reflect the realities of future deployment of HRI in industry or other environments, due to the differing social context.

### Manufacturing Environment (Non-unionised, Engaged Stakeholders)

As a follow-up, we sought to develop a more ecologically valid study by partnering with a local manufacturing firm and conducting a study on site with a manufacturing workforce [75].

The firm employed little automation, with only one standard manufacturing robotic cell, which was seen as a poor fit to the remaining operations. However, the firm was keen to adopt collaborative robots, which were seen as a better fit, to reduce safety risks to machine operators. The target process for robot deployment comprised of spot-welder operators assembling a metallic clamp and then welding the assembled components into the finished product. Given the hazards of working at speed with the welding equipment, the firm had an interest in deploying (comparatively safer) collaborative robots between the operator and spot-welder to reduce direct interaction with the hazardous welding equipment.

The recruitment process for this study relied on communication with staff via the firm; this substantially shaped the motivations for participation and people’s involvement with the research. From the outset, staff expressed interest in participation primarily due to their concerns regarding robots in their manufacturing process. Specific concerns included having to retrain to work with robots, the safety of the robots (operators had only encountered non-collaborative, caged industrial robots) and/or the risks of being replaced by robots. Volunteers saw participation as a means of preserving their job in a changing work environment.

Moreover, concerns were raised about the task itself, the data collected and how it would be used; the task consisted of repetitive manual actions requiring both speed and accuracy, which were seen as reflections of those on the factory line. Prospective participants were concerned that ‘poor’ performance in the experimental study could be used to inform redundancies or be otherwise applied to have negative consequences at their workplace. Despite the task being identical to that of Sect. 3.1 (and therefore dissimilar to participants’ tasks at work) the study population’s views towards the robot and the proposed interaction scenario were already much more negative. Potential participants were not expressing a negative attitude towards robotics per se, but a negative attitude towards how the Deployer (in their view, the manufacturing firm) might use robotics as a result of the study. The already limited trust between the participants and the Deployer of the HRI scenario would therefore shape the interaction itself.

Groundwork was undertaken to address the influence of the Deployer via a process of responsible innovation to include staff as engaged stakeholders in the research. Staff from the firm participated in focus groups and co-design workshops relating to the potential use of robotics in terms of safer working practices, and to the further development of the ISO-style signage [76]. These co-design processes enabled participants to visualise and consider potential HRI contexts and scenarios and prompted in-depth conversations on a variety of themes. Participants identified aspects of co-botic scenarios that could improve their personal safety and identified key matters regarding allocation of individual, team, and firm responsibility for a robot’s operation. Post-hoc qualitative evaluation described this as having ‘considerable’ positive impact on participants’ attitudes towards collaborative robotics [76] [p. 124].

Through the workshops and further tripartite discussion, the research team were able to assert their independence from the manufacturing firm; while the firm would host the HRI scenario the research team would function as the Deployer. Data collected from the study would only be made available in aggregate form and participation would be kept as anonymous as possible. While the firm would have to know who had participated as they were permitting the study to take place on-the-clock, they would not be informed about any individual’s performance or views expressed. Thus, initial concerns about trusting the firm with performance data were allayed by changing who participants saw as the Deployer. Crucially for the current work, this occurred without a change in the HRI scenario itself. Negative attitudes towards the robot could be addressed outside of Measured HRI as trust and attitudes were being (re)shaped at the Organisational Level.

### Manufacturing Environment (Unionised, Non-engaged Stakeholders)

The third study was set out as a direct replication of the second to examine the differences and similarities between manufacturing organisations. Where in Sect. 3.2 the firm was new to robotics, the aerospace firm here had developed a robotics system but was facing difficulties in deploying it due to staff concerns. Our aim for the study was to examine if these differences in readiness and extant views towards robotics would carry over to responses to the ISO style signage and performance at the task.

As with Sect. 3.2, there were challenges regarding recruitment, however, in this instance these proved substantial enough to prevent the study ever getting off the ground. First, the proposed study was seen within the context of a pre-existing dispute between the firm and the union representing the manufacturing workforce. Given the dispute regarded automation and use of robotics, any assurances of independence in conducting the research were immediately dismissed; unlike the firm in Sect. 3.2, this firm’s management were seen as the sole Deployer. Where concerns were raised about the firm in Sect. 3.2 targeting employees who had ‘underperformed’ in the task, in this there was concern that the study itself would pave ways for across-the-board changes which were unwanted. Second, direct engagement with the workforce in the style of the prior study was not considered achievable by management, and so the practices which had successfully demonstrated independence and engaged the workforce as stakeholders in the research could not occur. On their part, management raised concerns that participant responses were more likely to reflect attitudes towards the firm than the HRI scenario. This evident breakdown of trust between potential Users and the Deployer rendered any study of trust towards the Robot moot.

In this case, individual perceptions towards the robot and performance in the co-botics task are obviously impossible to determine. That said, this study’s failure to launch highlights the contextual effect of trust in the Deployer upon trust towards robotics, which in was constrained at the Organisational level. Any data (including the existence of study itself) was seen by the manufacturing staff as being of potential use by the Deployer against the participants and the population from which they would be drawn. Thus, had the study gone ahead, *any* measurement taken may have been indicative of User trust towards the (perceived) Deployer in addition to, or even in place of, trust in the Robot.

### Summary

Across the three studies, the research team and the HRI task were the same, and yet they were remarkably different in their unfolding. Were we to focus on Measured HRI only, it might seem that this arose from individual differences, (e.g., experience with manufacturing and automated systems) affecting attitudes towards HRI. In particular, the machine operators’ experiences, including stories of ‘jumpy’ or ‘temperamental’ welding tools that caused delays and injuries, could be associated with greater trepidation towards HRI than that of the university population, whose unfamiliarity could equally lead to a cautious or more blithe approach. However, the concerns raised in the manufacturing environments were based at the Organisational rather than Physical Level: a lack of trust not towards the robot but towards management. While individual differences do play their part in shaping trust towards robotics [8], recognition of the role the Deployer plays in shaping HRI scenarios offers a substantive explanation for the outcomes.

## Care Robotics Focus Group

As we addressed earlier, there are two cornerstones of social robotics research: the simulation of social processes in robotic agents, and the study of people’s social processing and experiences regarding HRI. Both dynamics invite trustworthy HRI investigation. The studies outlined in Sect. 3 indicate potential influence from the Deployer on how participants may approach HRI: lack of trust towards a Deployer, particularly of the Deployer’s intentions, may present engagement and interaction issues with the HRI scenario. This work points to one aspect of trust in the triad - the relationship, and social processing, between User and Deployer. However, the robot (KUKA iiw) used in these studies is not designed to simulate social processes, and is thus not readily considered a ‘social robot’, although, arguably, it does exist as a social presence in its role as an agent in a behavioural task. It is important to establish whether HRI measures concerning social trust are particular to social robotics, or more generally apply to interactions with robotic agents within deployment contexts (be they industrial or healthcare related, or something else). Given this, we here present work to further explore the pathway between User and Deployer via robots specifically designed to be social. In this case, findings from a focus group-workshop on health-social care robotics.

The workshop used the LEGO^®^ Serious^®^ Play (LSP) method [77] to investigate people’s ‘sociotechnical imaginaries’ - projections of a collectively achieved future, brought about by advances in technology [78]—in the use of robots for health-social care. Full details of the workshop are reported elsewhere [79] and summarised here.

### The Workshop

Eleven participants (ages between 18–30; 5 male and 6 female) from the University’s opt-in research volunteers list joined the two hour in-person workshop. The LSP workshop began with a series of warm-up exercises to familiarise participants with using the LEGO bricks as metaphors and that they may imbue pieces or whole models with their own meaning (e.g., a green square may signify a plant, growth, green-energy, envy...).

The core of the workshop began with participants building and then describing a model’s story in which a robot provides care to someone; both ‘robot’ and ‘care’ were left intentionally undefined to explore scenarios that immediately came to mind. Following this round of building, participants were asked to rebuild their model to invert their story’s tone (i.e., complicate positive stories or improve uncomfortable ones). This aimed to draw out the key issue in each story and which aspect of robotics deployment this related to (e.g., robotic design, regulation, interaction context).

Video and audio was recorded throughout for transcription and for referral back to the models constructed. Participant discussions were anonymised, transcribed, and analysed using the software package Nvivo 11. Two authors individually open-coded participants’ explanations of the models and subsequent discussions into first-order codes, which, following discussion between authors, were grouped into five aggregate themes.

### Findings

Overall, participants imagined a wide range of robots performing an equally diverse range of functions embodying different aspects of ‘care’; examples include robots supporting care home staff with physical tasks through to personalised daily care (such as providing advice and assistance with the day’s grooming and wear). Across stories, the relationship between robot and user was commonly first envisioned as reciprocal and supportive, with the robot acting semi-autonomously to determine effective means of meeting user needs.

Though this study of eleven participants is too small to expect thematic saturation, five aggregate and interlinked themes of Trust, Comfort, Necessity, Dependence and Control were apparent. The related themes of Trust and Control are discussed here.

#### Trust

Participants built scenarios representing trust as an interpersonal quality, including views that an idealised care robot would not be judgmental and thus could be trusted with users’ weaknesses and vulnerabilities. Specifically, this ranged from a robot being trusted to not make adverse judgements of one’s messy house through to a robot serving as confidant for embarrassing or sensitive problems. Participants collectively concluded that it would be ‘*easier to ask the robot to be confidential about your information than to ask someone to keep the confidence*’ (Person5). This value of trusting a robot to keep confidence is further seen in a scenario envisioning a social robot for supporting the mental and physical care of company employees, ‘*in the [competitive] working environment people barely share their emotions, barely share their problems*’ (Person6).

Of note is the apparent independence of the imagined robots, namely that they are able to keep confidence. When asked to invert the care scenarios, participants identified concerns on how a robot keeping confidence would work in practice. Person5 identifies ‘*[the robot] will realise everything... So maybe someone will take advantage of this kind of knowledge, this kind of information about the staff, like the manager... this is a serious problem of my robot*’. In effect, trust towards a robot with ones vulnerabilities can only really be extended as far as trust towards a Deployer with access to and/or management of the robot.

#### Control

Similar concerns were raised in terms of Control: other people (be they the intended Deployers or malicious actors) could potentially access information gleaned during HRI, which may then be turned against the Users. Further concerns related to robots identifying one’s weaknesses or vulnerabilities through interaction, that could be made apparent to Deployers in workplace settings. Person3 states ‘*But these [nursing assistant robots] are all over here in the staff room complaining that they’ve basically been designed to be as efficient as possible but the nurse keeps getting in the way of them being able to do their job by trying to talk to patients and care for them*’. In effect, User interaction with the robotic agents becomes another means by which the Deployer (in this case, management at the User’s workplace) can monitor behaviour, potentially shaping interaction itself.

There is further recognition of any care robots provision necessarily requiring their integration into systems with human oversight to enable human-centred care, ‘*not just leaving [robots] to their own devices but having people behind the scenes so it’s not just completely lacking humanity*’ (Person6). The complexity of the overall narratives poses ‘control’ as situated across the relations within the Triad: between Robot and User, Deployer and User, and Deployer and Robot. The initially-positive stories detailing care robots as independent agents unravel when inverting the stories’ tone; specifically, participants independently identify where Deployers rather than Users would hold control over these.

## Discussion

The exploration of trust and trustworthiness in HRI is a still emerging area of study. After a discussion of trust measurements and challenges as they pertain to HRI, we argue here that trust measures between a User and a Robot need to be mindful of the other relationships within the specific HRI context being studied. Users’ views towards a Robot can be influenced by the perceived trust of the individual or institution deploying the robot for use (the Deployer), as well as the perceived dynamic between the Deployer and the Robot.

Our case study indicates that this can occur even when the robot is not specifically designed to be social. It is also relevant in instances where the robot has a social presence born of involvement in behavioural interactions, contextually bound to people’s social processing, as can be the case in industrial HRI as well as in care-robotics. We evidence this argument by leveraging The Tech Ladder [37] to recognise concerns towards HRI expressed at an Organisational level as being distinct from Physical and Psychological Levels and as capable of shaping interaction. In terms of the case study, these concerns set out conditions under which people were willing to participate in interaction, if at all.

The case study further highlights that it is not necessarily the relationships and interactions with the social triad shaping trust, but rather the User’s *perceptions* of these. Despite the researchers functioning as the Deployer through (1) owning and programming the robot, (2) devising and overseeing the interaction scenario, and (3) collecting and handling the data, the manufacturing employees primarily saw their respective employers and management teams as the Deployer. In their view, the firms were responsible for the robot’s introduction and the oversight of the employees’ interaction with the robot. Where this view could be addressed in one instance (Sect.3.2) through a change in who was seen as the Deployer[76], that this was not seen as a viable route for engagement in another (Sect. 3.3) suggests further complexities of the Deployers’ role in HRI. Structured exploration of the role the Deployer may have in shaping HRI is recommended and suggestions for this are presented in Sect. 5.2.

Abstracting away from our industrial contexts, outcomes from our care-robotics focus group indicate that even within a framework explicitly designed around interactions with a social robot, similar outcomes were observed. Users initially depicting robots they could intimately trust on a social basis later became conscious of how broader dynamics between themselves, the robot, and the individuals or institutions deploying those robots (User-Robot-Deployer) would influence how far they could trust *their* social robot.

Despite the remit of the focus group placing few, if any bounds on what ‘robot’ or ‘care’ meant for the participants, their imaginaries still brought forth complicating aspects of a Deployer in some way overseeing, or potentially making use of information gleaned from any HRI taking place. Users imagining themselves owning and/or programming a robot, and devising and overseeing their care-based interaction scenarios, still saw that trust in an interaction with *their* robot became mediated by an *imagined* management. Their questioning where to place confidence depending upon where their information was held, and wondering what scenarios would lead to being taken advantage of, is indicative of the question: What does trusting the robot truly entail?

The change seen in Sect. 3.2 suggests a mechanism by which the Deployer may affect trust in HRI. In research on human-human interaction, trust is often represented as an individual’s revealing of a vulnerability during interaction, and the belief that another agent will not exploit this [80]. That second agent, now aware of the vulnerability potentially holds power over the first.^4^ The Deployer, by nature of controlling the design of the scenario, directing the User in their engagement with the scenario, and determining the measurements taken and how such measurements are used, has relatively high power in the social triad (cf. The Authority [35]). Comparatively, the User, less able to shape the bounds of the scenario and potentially not even the range of actions possible within the scenario holds less power. Thus it is clear that the Deployer, though external to the immediate HRI scenario, is nonetheless still present in shaping the interaction and any measurement of trust taken therein.

In terms of Sects. 3.2 and 4.2.2, employees and participants raised concerns regarding their vulnerability in participating, and questioned how information about their performance, or imagined monitored interactions, could be used by those who hold power over them (e.g., their employer or care staff).

Most explicitly in Sect. 3.2 (particularly in comparison to Sect. 3.1) we see that absent of any information indicating otherwise, participants’ assessment of the employer as the potential Deployer, and one that would not be trusted with vulnerability, served sufficient risk to distrust the entire scenario. Demonstration of the research team’s independence and subsequent inclusion of the employees within the development of core materials for the study, re-balanced power such that they were willing to participate [76]. As stakeholders in the work, and with greater degree of control over the scenario (in ways that the research team now relied upon) the social triad for Sect. 3.2 could approach the more agreeable dynamic of the User and Deployer holding comparable levels of power and investment in the interaction.

### Current Limitations

The Social Triad Model still remains to be formally tested; the studies described in this paper serve as an emerging evidence base used in developing the model and highlight the potential influence a Deployer may have on HRI, both in terms of interaction itself and in researching interaction. Similarly, the complexities apparent across the case study wherein co-design workshops were sufficient to shape perceptions around the Deployer for one instance, but not another, suggest this is not just a matter of objectively identifying the Deployer. User perceptions of the Deployer, including *who the Deployer even is* suggest further work for researchers in establishing the views of Users regarding Deployers and their relationship, as well as with the robot as part of HRI scenario design.

The work so far has examined trust with a view towards elevating trust; there is an emerging body of new research examining the risks of over-trust of robots and means to successfully reduce reliance and trust where appropriate (e.g., [82, 83]). This may present separate challenges to those currently discussed, particularly in navigating a decline in trust towards the robot, while still presumably maintaining trust towards the Deployer.

While the field currently has specific and operationalized measures of trust in HRI, these relate to the interaction between the user and robot only. This paper has not addressed means by which to formally record and measure the relationship between Deployer and User, nor the influence of the Deployer on HRI, let alone disentangle these influences; nonetheless, this paper brings to light the Deployers’ influence in the first place. With recognition of the potential influence of the Deployer, we can now propose a series of research directions and specific studies to examine their actual influence.

### Future Directions

There are many ways by which the Social Triad model can be formally explored in HRI studies, both specifically relating to social robotics research and research relating to the wider social context of the deployment of robots. The model also offers new context for any existing research that sees differences in findings between ‘lab-based’ HRI scenarios and those same scenarios in place in the field.

A substantive step forward would be the development of a new HRI trust scale that incorporates the role of the deployer in HRI. Potential avenues for this could come in the form of adaptations made to existing HRI scales that approach trust’s social context (e.g., [19]) or through the adaptation of scales used in other fields that explore similar relationship dynamics between user and deployer, such as those used to measure manager-employee trust [84]. Whether there is a ‘best’ approach to this - of building up from HRI scales or working down from human-human trust scales - remains an open question but one that itself may shed light on how HRI resembles and differs from human-human interaction through finding new ways to account for human and social contextual influences in HRI scenarios.

Empirical studies on the effects of the Deployer on user trust towards robots can be conducted in the field and lab based on the experiences we have observed in our case study. By measuring variations in trust towards the Deployer, structural models could be constructed to examine the influence this has on trust towards their robots. Specific experimental manipulations can be made of how the Deployer is presented in studies to gauge their influence on HRI. Varying the apparent institution responsible for deploying the robot (e.g., the hypothetical deployment of an emergency response robot by the more-trusted National Health Service versus the less-trusted police service [85]) may affect trust towards the robot deployed. Alternatively, variations in the Deployer’s apparent intention or motivations for use of a robot (e.g., in a manufacturing setting, use of a robot to increase safety versus to increase throughput) may communicate differing degrees of benevolence and competence of the deployer, indirectly shaping trust towards the robots used.

Further alternatives could examine contexts wherein the Deployer-User trust relationship is an established variable. For example, the relationship between a physical therapist (PT) and their patient is heavily mediated by trust [86]. By extension any tool, robotic or otherwise, deployed by the PT is reliant upon the trust the User has in their clinician. The use of complex non-social robots in clinical physiotherapy could offer an excellent starting point. PT robotics can be understood as sitting between industrial robots deployed as tools in a workplace, and social robots deployed in caring roles (as in [87]). Such HRI studies framed around already complex human-human trust dynamics will allow us to tease out where the trust lies between a robot and the person using the robot as a tool with the user. This could help establish new baselines for measuring trust in HRI which consider the Deployer as a vital variable in the collection and analysis of data.

The implied agency in autonomous behaviour from social robots offers further potential to examine Deployer effects through manipulation of the Deployer-Robot connection. Greater distancing between these, such as a robot asserting through synthetic social means a confidentiality of interaction and apparent independence from the Deployer, may moderate Deployer effects in the Social Triad. In sum, the Social Triad Model increases the number of possible interactions and highlights those not ordinarily accounted for. Based on the experience across the Case Studies and the liminal nature of a social robot as independently social, but not an independent agent, we offer the following specific predictions:

1. A Deployer that Users are suspicious of would result in a lower-trust interaction scenario than one they consider more trustworthy, even if the interaction itself is identical.
2. Participant’s articulation of views towards the Deployer would explain some variation in User-differences in interactions that are otherwise normally attributed to factors such as experience with robotics and demographics.
3. A social robot’s apparent agency and independence from the Deployer may moderate Deployer effects on trust.
4. Attempts to scaffold trust through the Robot enacting social behaviours (e.g., apologies for mistakes) may have a counter-intuitive negative impact on perceived trustworthiness in scenarios where Users do not trust the Deployer.
5. Inclusion of the User in HRI scenario development and/or empowerment of the User over the scenario could promote trust in the Deployer and, indirectly, the Robot.

It is clear that comprehensive research on trust in HRI is a growing and vital area. Our Social Triad Model, with the inclusion of the Deployer, is one route to better understanding, and overcoming, barriers that exist to effective and efficient trustworthy robotics.

### Practical Impact and Wider Implications

The apparent agency of a robot and the independence from the Deployer that this implies, especially for robots with their own ‘personality’ and social behaviours, presents a dilemma for recent models of trust that include social contexts. Where social interaction from a robot can influence trust [14–18, 46], robots, as yet, have no capacity to *experience* the social and emotional processing these involve. This highlights the distinction between the simulation of social processes in robotic agents, and people’s social processes with ‘things’ they engage with in the world that have social presence irrespective of the ‘thing’s’ purpose (especially when those ‘things’ are a proxy for another human as can be the case in HRI).

There are wider issues regarding the ethics of persisting with developing mechanisms for trust towards robots, especially within the social context [57]. As with other developments in autonomous technologies, it is worth considering the potential adverse consequences for the use of synthetic social strategies to gain user trust. Given the range of studies evidencing that trust can be affected through synthetic social behaviors - coming with little to no consequence for the robot or deployer - there is potential for use of these or similar to shape trust, beyond the original researchers’ intentions. If researchers (individually or collectively) were to find highly effective processes for robot social interactions to evoke trust, misuse by untrustworthy actors could quickly render those findings obsolete. Research into trust towards social robotics from a social context may then be better served to understand and explore the psychological phenomena of trust itself rather than as a means to the end of increasing trust towards robots.

Critiques highlight this hollowness of robots’ communication to not experience, and lacking genuine independence from Deployers [35, 43], as undermining social robotic interaction, or even as indicative that explorations of trustworthy robotics have little place outside explicitly social-robot-human interaction studies. Moreover, where Deployers are distrusted, a robot’s social interactions for trust could be regarded as manipulative or coercive actions to constrain users within the Deployer’s framework for the HRI scenario. By example from a neighbouring field, motivated by apologies from corporations following scandal or disaster, may be driven by intent to restore customer loyalty rather than any sincere attempt to make amends [88, 89]. A robot’s synthetic social behaviours to garner trust perhaps should be considered as indirect expressions from the Deployer rather than the robot itself - that these communications are how the Deployer seeks to be *regarded* by the user. With this in mind, actions and synthetic social behaviours from robots may communicate across multiple channels of trust: trust of a robot’s competence at a task, trust of the Deployer’s competence in managing the robot, trust of the Deployer’s intentions in deploying the robot (i.e., their, rather than the robot’s benevolence).

To conclude, with the development of the Social Triad Model, we urge the field to consider the wider social context for any HRI scenario developed. With the increasing evidence that a robot’s synthetic social behaviors can affect user trust, it is vital to understand the processes behind this, the actors at play, and the consequences of affecting trust in this manner. Where successful social interactions affecting trust are mediated explicitly from non-social agents (Robots), should instead inspire a search for, and evaluation of, the social agents responsible (those Deployers responsible for the Robots). *In sum, to create trustworthy robots, the deployers themselves must become worthy of trust*.

## Supplementary Information

Below is the link to the electronic supplementary material.

Supplementary file 1 (pdf 249 KB)

## Data Availability

The data that support the findings of this study are available from the corresponding author upon reasonable request.

## Footnotes

1 https://www.tas.ac.uk.

2 In fairness to Malle and colleagues, we have also found participants occasionally respond with ‘does not apply’ when considering dimensions of integrity or warmth [32].

3 Comparisons of outcomes in terms of performance and attitudinal differences between studies are available in the Online Appendix, but are tangential to the current work.

4 Expressions of vulnerability by robots has also been demonstrated to shape trust towards them [81].
