# Supplementary material for: The Social Triad Model: Considering the Deployer in a Novel Approach to Trust in Human–Robot Interaction
Source: Int J Soc Robot. 2023 Sep 13;16(6):1405–18. doi: 10.1007/s12369-023-01048-3 (PMC11842407; doi:10.1007/s12369-023-01048-3)
Supplement: Supplementary file 1 — (pdf 249 KB) [file 12369_2023_1048_MOESM1_ESM.pdf]

This appendix provides a comparison of experimental outcomes from the two completed studies as described in the Deployer Case study. Full details of the individual studies for Study 1 (University population) and Study 2 (Manufacturing population - non-unionised, engaged stakeholders) are already available [1, 2] and summary information relevant for the comparison between studies is presented here.

Participants

Ninety participants took part in Study 1; of these, 30 completed the task with task-relevant static graphical signage (experimental condition), 30 completed the task with task-irrelevant static graphical signage (active control), and 30 completed the task without any static graphical signage (passive control). Forty participants took part in Study 2; 21 completed the task with dynamic graphical signage (experimental condition) and 19 completed the task without (passive control). Comparisons between the studies are made for the experimental and passive control conditions only as there is no active control condition for Study 2.

Measures

The following measures were collected across both studies: Task Accuracy (Successful trials as a percentage of all trials attempted); Mean Response Time (between bolt extractions), and the 'Operation of the Robot' subscale from the Negative Attitudes towards Robots Scale (NARS).

Procedure

In both studies, participants completed preliminary measures of NARS before interaction with the KUKA iiwa robot arm. They then completed the 'bolt extraction task' through direct manipulation of the arm; following the instructions provided: 'On the table in front of you, there are 16 narrow tubes; six of these contain M5 bolts. These bolts need to be collected, however they are inaccessible to people, and, although the robot can reach and pick them, it cannot identify which tubes contain bolts. You can spend up to 10 min on this task'. Participants were informed that their speed and accuracy would be measured. Last, participants completed post-interaction measures of NARS.

Results

As both studies used a between subject deign, a comparison was performed with one-way ANOVA. Results for Accuracy showed a significant difference between the studies ($F(3, 98) = 4.12$, $p = .009$). The post hoc analysis revealed a significant difference between the control groups only ($p = .021$). There was a also significant difference between studies in Response Time $F(3, 98) = 2.80$, $p = .044$; overall, participants in Study 1 were significantly slower than participants in study 2. Post hoc analysis did not indicate further differences between participant groups ($p > .483$).

The analysis on the NARS scores was conducted with a mixed ANOVA (before and after the interaction with robot as a function of the experimental condition). Neither main effect of NARS, nor interaction of NARS x condition were significant. The main effect of condition was significant, $F(1, 95) = 669.45$, $p \leq .001$. The pairwise comparison indicated that there were significant differences in almost all comparisons: the static experimental vs static control ($p \leq .001$), static experimental vs dynamic control ($p \leq .001$), dynamic experimental vs static control ($p \leq .001$), dynamic experimental vs dynamic control ($p \leq .001$).

Table 1 Accuracy, response time and NARS scores by study and participant group

		Accuracy		RT(s)		pre-test NARS		post-test NARS	
		M	SD	M	SD	M	SD	M	SD
Study 1	Experimental	.65	.26	7.46	3.21	11.63	4.21	11.83	4.53
	Control	.49	.27	8.41	4.28	1.83	3.00	1.83	3.26
Study 2	Experimental	.67	.18	7.51	2.31	6.67	3.20	7.22	4.07
	Control	.71	.20	7.25	3.67	6.00	2.61	5.29	2.03

Discussion

When interpreting the analysis of these two studies one need to keep in mind different populations of the trials (student and staff vs. manufacturing operators) and the changes in the stimuli between studies (static vs dynamic signage). Further consideration should be made to the fact that the manufacturing operators (i.e., Study 2 participants) had recently participated in a workshop specifically designed to address negative attitudes towards robotic operation [3], which was not available to university staff and students (i.e., Study 1 participants).

Results indicate some small differences in performance between participants, across studies; as would be expected given their experience in related working processes, manufacturing operators have greater accuracy and faster response time than university staff and students.

Study 1 participants have a much wider variation in their NARS scores than the Study 2 participants, but overall differences are not significant. Again, this may be due to the manufacturing operators greater experience (consistent across these participants) with related working processes, whereas unfamiliarity with human-machine interaction could lead to worry or blithe acceptance. Of note, there are no differences in the *changes* to NARS scores across the studies or conditions, despite finding significant changes within conditions following the HRI scenario. Results broadly indicate that the initial and substantial concerns raised by Study 2 participants regarding robotics, which were addressed in the co-design workshop [3], are not reflected in the data when it came to participation.

[1] Eimontaite, I., Gwilt, I., Cameron, D., Aitken, J.M., Rolph, J., Mokaram, S. and Law, J.: Language-free graphical signage improves human performance and reduces anxiety when working collaboratively with robots. The International Journal of Advanced Manufacturing Technology **100**(1), 55-73 (2019)

[2] Eimontaite, I., Cameron, D., Rolph, J., Mokaram, S., Aitken, J. M., Gwilt, I., & Law, J.: Dynamic graphical instructions result in improved attitudes and decreased task completion time in human–robot co-working: an experimental manufacturing study. Sustainability **14**(6), 3289 (2022)

[3] Gwilt, I., Rolph, J., Eimontaite, I., Cameron, D., Aitken, J., Mokaram, S., Law, J.: Cobotics: developing a visual language for human-robotic collaborations. In: Cumulus Conference Proceedings Paris 2018 – To Get There: Designing Together, pp. 106–126 (2018)
